# Role of adhesion forces in mechanosensitive channel gating in *Staphylococcus aureus* adhering to surfaces

**DOI:** 10.1038/s41522-020-00141-z

**Published:** 2020-08-21

**Authors:** Vera Carniello, Brandon W. Peterson, Henny C. van der Mei, Henk J. Busscher

**Affiliations:** Department of BioMedical Engineering, University of Groningen and University Medical Center Groningen, Groningen, Netherlands

**Keywords:** Sequencing, Antimicrobials, Bacteria, Biofilms

## Abstract

Mechanosensitive channels in bacterial membranes open or close in response to environmental changes to allow transmembrane transport, including antibiotic uptake and solute efflux. In this paper, we hypothesize that gating of mechanosensitive channels is stimulated by forces through which bacteria adhere to surfaces. Hereto, channel gating is related with adhesion forces to different surfaces of a *Staphylococcus aureus* strain and its isogenic Δ*mscL* mutant, deficient in MscL (large) channel gating. Staphylococci becoming fluorescent due to uptake of calcein, increased with adhesion force and were higher in the parent strain (66% when adhering with an adhesion force above 4.0 nN) than in the Δ*mscL* mutant (40% above 1.2 nN). This suggests that MscL channels open at a higher critical adhesion force than at which physically different, MscS (small) channels open and contribute to transmembrane transport. Uptake of the antibiotic dihydrostreptomycin was monitored by staphylococcal killing. The parent strain exposed to dihydrostreptomycin yielded a CFU reduction of 2.3 log-units when adhering with an adhesion force above 3.5 nN, but CFU reduction remained low (1.0 log-unit) in the mutant, independent of adhesion force. This confirms that large channels open at a higher critical adhesion-force than small channels, as also concluded from calcein transmembrane transport. Collectively, these observations support our hypothesis that adhesion forces to surfaces play an important role, next to other established driving forces, in staphylococcal channel gating. This provides an interesting extension of our understanding of transmembrane antibiotic uptake and solute efflux in infectious staphylococcal biofilms in which bacteria experience adhesion forces from a wide variety of surfaces, like those of other bacteria, tissue cells, or implanted biomaterials.

## Introduction

Mechanosensitive channels in bacteria are located in the bacterial lipid membrane surrounding the cytoplasm and are composed of multiple protein subunits^1^. Mechanosensitive channels allow bacteria to interact with their environment and act as valves that open or close in response to changes in membrane tension^2–4^. This allows solutes to rapidly flow across the membrane due to a decrease in environmental osmolarity or hypo-osmotic shock^5,6^ that can both generate membrane tension changes^7^. It has been suggested that gating events play a role in the release of autoinducers to stimulate signaling events that are important in the genesis or maintenance of a biofilm^8^. The most studied types of mechanosensitive channels are the mechanosensitive channels of large (MscL)^9^ and small (MscS) conductance^10,11^. Opening of MscLs in *Escherichia coli* yielded pores with a diameter of about 30 Å^12^ requiring a critical membrane tension^13^ of around 10 mN m^−1^, while physically different MscSs required a lower tension^14^ of about 5 mN m^−1^ to open pores of about 16 Å^13^. However, mechanosensitive channels do not only function to protect a bacterium against osmotic forces, but also have been suggested as well to play a role in antibiotic uptake next to their role in solute efflux^15,16^. MscLs provide the main pathway for bacterial uptake of the antibiotic dihydrostreptomycin^17^, which is a large molecule that can induce MscL opening to promote its uptake^15^. Low doses of dihydrostreptomycin inhibit the growth of wild-type bacteria having MscLs, but do not significantly inhibit Δ*mscL* mutants lacking MscLs^15^. Moreover, MscLs are also responsible for efflux of potassium^15^ or cytoplasmic proteins^18,19^.

The role of mechanosensitive channels in antibiotic uptake and solute efflux by bacteria makes a better understanding of the nature of the forces that can lead to the channel opening or closing imperative, particularly in an era of increasing antibiotic resistance among many pathogenic bacteria, including *S. aureus*. The majority of bacterial infections arise from bacteria in a biofilm-mode of growth in which bacteria adhere either to each other, to mammalian cells, to mineralized tissue such as bone or teeth, or to biomaterial-implant surfaces. This triggers the hypothesis that opening or closing of mechanosensitive channels cannot only be triggered by osmotic forces, but also by the forces through which bacteria adhere to a surface^20^, as in the first stage in biofilm formation^21^. Surface enhanced fluorescent imaging of bacteria adhering to metal surfaces has clearly shown that adhesion is accompanied by nanoscopic deformation of the bacterial cell wall^22^. Cell wall deformation continues until the reaction forces arising from the relatively rigid peptidoglycan layer equal the adhesion forces experienced by an adhering bacterium. Δ*pbp4* mutants of *Staphylococcus aureus* deficient in peptidoglycan cross-linking, accordingly showed a faster cell wall deformation upon adhesion than its parent strain with cross-linked peptidoglycan^22^. The forces by which bacteria adhere to a surface and induce cell wall deformation, will affect lipid membrane tension in adhering bacteria, equally as osmotic forces affect membrane tension in planktonic ones. This forms the basis of our above hypothesis that bacterial adhesion forces may impact the gating of mechanosensitive channels^23^.

The aim of this paper is to provide evidence in support of the hypothesis that the adhesion forces experienced by bacteria adhering to a surface, play a role in the opening and closing of mechanosensitive channels, next to other established driving forces. Although Gram-negative *E. coli* are often used for the study of mechanosensitive channel gating^3,9,23–27^, we chose to use Gram-positive *Staphylococcus aureus*. *S. aureus* is a common pathogen, increasingly involved in multi-drug resistant infections including notoriously hard to treat biomaterial-implant associated infections^28,29^. We assessed mechanosensitive channel gating by measuring uptake of fluorescent calcein (MW 623 Da) and the antibiotic dihydrostreptomycin (MW 731 Da) in staphylococci adhering to different substratum surfaces. The forces by which staphylococci adhered were measured using single-bacterial contact probe atomic force microscopy (AFM). Gating in *S. aureus* RN4220 adhering on different substratum surfaces was compared with the gating in an adhering, isogenic Δ*mscL* mutant, deficient in MscL conductance.

## Results

### Physico-chemical characterization of substratum and bacterial cell surfaces

Physico-chemical characteristics of the substratum surfaces used, as well as of the parent strain, *S. aureus* RN4220, and its isogenic Δ*mscL* mutant were first evaluated. Substratum surfaces were evaluated with respect to their roughness and hydrophobicity, which are both important characteristics of a surface governing adhesion forces^30^. Substratum surfaces had slightly different roughnesses between 3 nm for gold to 13 nm for polystyrene (Table 1). These roughnesses can be considered sufficiently smooth not to influence contact angles with liquids^31^, which we used to measure the hydrophobicity and surface free energies of the substratum surfaces. Water contact angles were significantly (*p* < 0.001, *F* = 463, df = 17) smaller on glass and glass with a Pluronic-coating, as compared to more hydrophobic polystyrene and gold. The differences in contact angles among the three liquids employed led to higher electron-donating parameters and total surface free energies for glass and Pluronic-coated glass, as compared to polystyrene and gold (Table 1).

**Table 1:** Physico-chemical surface characteristics of the substratum materials.

	Polystyrene	Gold	Glass	Pluronic-coated glass
Roughness (nm)	13 ± 5	3 ± 1	4 ± 1	8 ± 3
*θ*_w_ (°)	80 ± 2	82 ± 3	10 ± 1	9 ± 3
*θ*_f_	64 ± 2	65 ± 4	10 ± 1	14 ± 4
*θ*_m_	35 ± 1	54 ± 2	34 ± 3	45 ± 2
γ^−^ (mJ m^−2^)	10 ± 4	5 ± 2	55 ± 1	56 ± 2
γ^+^	0 ± 0	0.3 ± 0.4	0.9 ± 0.2	2 ± 0.1
γ^AB^	0 ± 0	2 ± 1	14 ± 1	19 ± 0.4
γ^LW^	42 ± 0.3	32 ± 1	43 ± 1	37 ± 1
γ^tot^	42 ± 0.3	33 ± 2	57 ± 0.2	56 ± 1

Surface roughness, contact angles measured with water (*θ*_w_), formamide (*θ*_f_), and methylene iodide (*θ*_m_), and surface free energy parameters and components. Total surface free energy γ^tot^ results from Lifshitz–Van der Waals γ^LW^ and acid-base γ^AB^ components, and electron-donating γ^−^ and electron-accepting γ^+^ parameters. ± signs represent standard deviations over measurements on three different substratum surfaces.

**Fig. 1:**
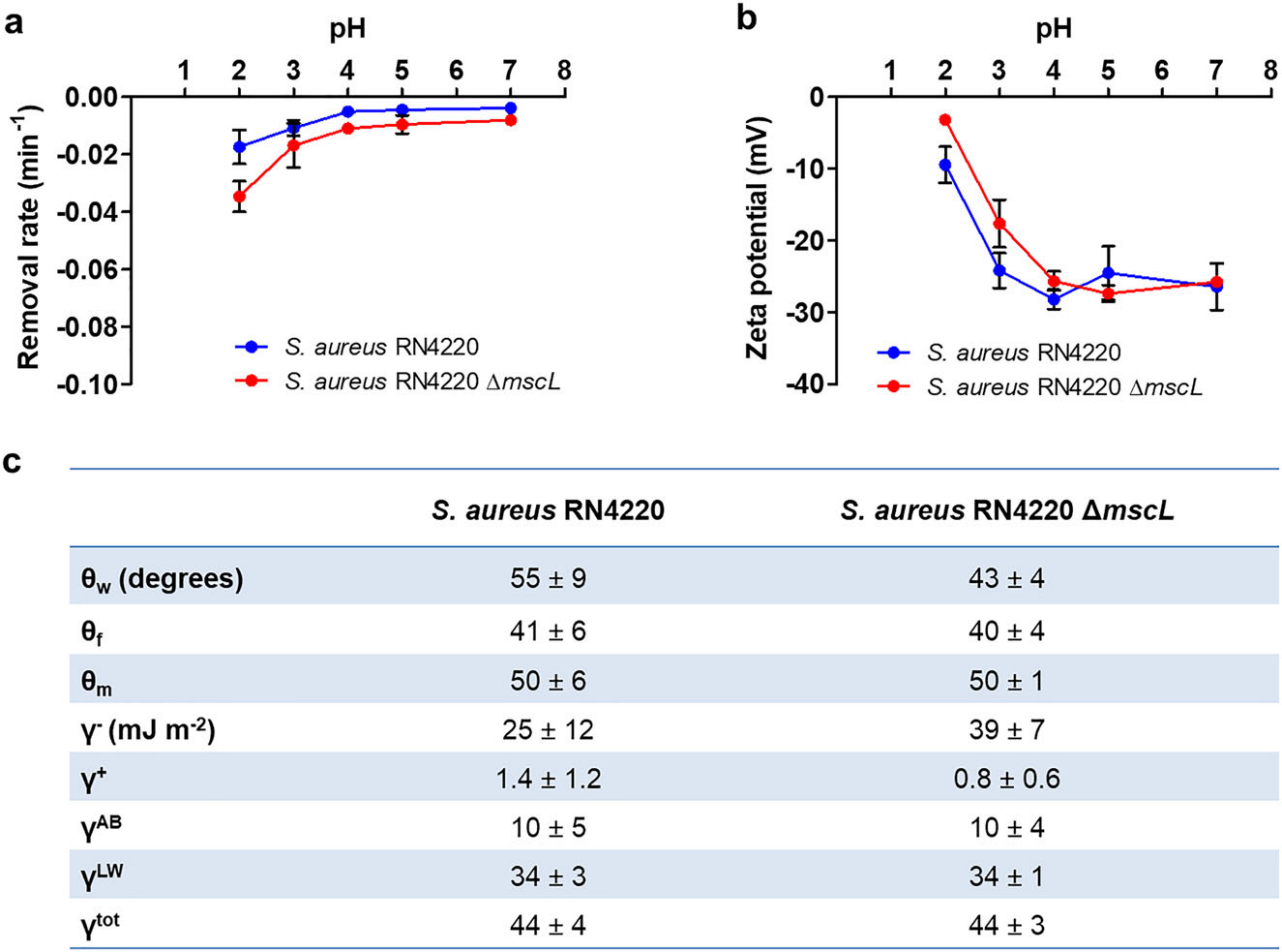
Physico-chemical characteristics of staphylococcal cell surfaces. **a** Initial bacterial removal rates from an aqueous phase (10 mM potassium phosphate buffer) by hexadecane as a function of pH. **b** Zeta potentials of staphylococci in 10 mM potassium phosphate buffer as a function of pH. **c** Contact angles on bacterial lawns with water (*θ*_w_), formamide (*θ*_f_), methylene iodide (*θ*_m_), and staphylococcal surface free energy parameters and components. Total surface free energy γ^tot^ results from Lifshitz–Van der Waals γ^LW^ and acid-base γ^AB^ components, and electron-donating γ^−^ and electron-accepting γ^+^ parameters. Error bars in panels (**a**) and (**b**) represent standard deviations over measurements on three different bacterial cultures. ± signs in panel (**c**) represent standard deviations over measurements on six bacterial lawns, prepared from three separate bacterial cultures.

Both *S. aureus* strains showed extremely small initial removal rates from an aqueous phase by hexadecane (Fig. 1a), which indicates either a hydrophilic bacterial cell surface or electrostatic double-layer repulsion between staphylococci and hexadecane, or both^32^. To assess the contribution of electrostatic double-layer interactions to staphylococcal removal by hexadecane, bacterial zeta potentials were measured as a function of pH (Fig. 1b). Both strains had negative zeta potentials between pH 2 and 7. Note that bacterial zeta potentials were closer to zero toward pH 2, concurrent with a slight increase in removal rate by hexadecane due to decreased electrostatic double-layer repulsion. At pH 7, used in our gating experiments, both strains had a similar (two-tailed Student’s *t*-test, *p* = 0.7415, *t* = 0.3535, df = 4) zeta potential of −26 mV (Fig. 1b). Water contact angles on bacterial lawns indicated that both strains were hydrophilic with water contact angles <60°. Surface thermodynamic analysis of the contact angles measured with the three different liquids on each bacterial strain, generated a higher electron-donating parameter (γ^−^) for the Δ*mscL* mutant compared to the parent strain (*p* = 0.0254, *t* = 2.551, df = 12), and minor differences (Fig. 1c; *p* > 0.05) in other surface free energy parameters and components, ultimately resulting in a similar total free energy for both strains (Fig. 1c; *p* = 0.8941, *t* = 0.1359, df = 12).

In line with these physico-chemical characteristics of the parent and mutant strains, initial adhesion forces of the different staphylococci, i.e., measured before bond-maturation, were comparable (Table 2). Taken together, the physico-chemical characteristics of the surfaces of *S. aureus* RN4220 and its isogenic mutant can be considered highly similar with negligible differences bearing no biological significance. This implies that the outermost cell surface in both staphylococcal strains is identical and that deletion of the *mscL* gene did not affect the outermost cell surface.

**Table 2:** Staphylococcal adhesion forces before and after 10 s bond-maturation.

	*S. aureus* RN4220 *F*_i_ (nN)	*S. aureus* RN4220 *F*_10 s_ (nN)	*S. aureus* RN4220 Δ*mscL* *F*_i_ (nN)	*S. aureus* RN4220 Δ*mscL* *F*_10 s_ (nN)
Polystyrene	0.4 ± 0.4	3.1 ± 2.3	0.3 ± 0.2	3.0 ± 2.4
Gold	1.1 ± 0.8	5.3 ± 2.7	2.5 ± 1.4****	20.4 ± 3.8****
Glass	0.9 ± 0.6	3.5 ± 2.4	0.6 ± 0.3	3.4 ± 1.9
Pluronic-coated glass	0.3 ± 0.2	1.6 ± 1.0	0.2 ± 0.2	0.5 ± 0.6***

Initial adhesion forces *F*_i_ between staphylococci and substratum surfaces, and adhesion forces after 10 s bond-maturation *F*_10 s_. ± signs represent standard deviations over at least 45 force–distance curves, comprising for one bacterial probe three different spots, recording five force–distance curves on each spot. Three probes were prepared from three separate bacterial cultures. *indicates significant differences from *S. aureus* RN4220 (Kruskal–Wallis test, ***p < 0.001, ****p < 0.0001).

### Bacterial adhesion forces

Adhesion forces between staphylococci and substratum surfaces were measured with single-bacterial contact probe AFM, allowing 10 s for the bond between adhering staphylococci and substratum surfaces to mature after initial contact. Among the different substratum surfaces, gold generated the strongest adhesion forces for both strains, while the weakest forces were recorded on Pluronic-coated glass (Table 2). Note that the range of force values observed over the different substrata after bond-maturation was relatively small in *S. aureus* RN4220 (1.6 nN on Pluronic-coated glass to 5.3 nN on gold), while being larger in the Δ*mscL* mutant (0.5–20.4 nN on Pluronic-coated glass and gold, respectively). Numbers of adhering staphylococci were low for both strains, corresponding with a bacterial coverage of the substratum surfaces of <5%.

### Uptake of calcein in adhering staphylococci

Gating of mechanosensitive channels in adhering and planktonic staphylococci was evaluated by exposing bacteria to the fluorescent dye calcein, and its transmembrane transport quantitated by enumerating the number of fluorescent bacteria, as previously described for liposomes^33^. To ensure similar action of calcein in staphylococcal gating as in its uptake by liposomes^33^, it was first assessed whether the different channels of small and large conductance were closed in planktonic staphylococci and calcein uptake was absent. Indeed, after staphylococcal exposure to calcein and subsequent additional cross-linking through fixation by formaldehyde^34,35^ to prevent calcein outflow, low percentages (3–7%) of fluorescent staphylococci were observed (Table 3). Also blocking through formaldehyde fixation of channels prior to calcein exposure to prevent calcein uptake, yielded a low percentage of fluorescent staphylococci (6–13%). Exposure of adhering staphylococci to calcein followed by formaldehyde fixation to prevent calcein outflow, yielded 45% fluorescent bacteria, both in the parent strain and the Δ*mscL* mutant. Inducing channel closure by altering lipid packing with gadolinium in adhering staphylococci^36^, as also common in liposomes^37,38^ and bacteria^39,40^ prior to exposure to calcein, prevented uptake of the fluorescent dye, as evidenced by similarly low percentages (6–9%) of adhering fluorescent staphylococci as in planktonic staphylococci (see also Table 3). Collectively, these data suggest that gadolinium and formaldehyde exposure limit transport through MscLs, albeit by different mechanisms.

**Table 3:** Validation of calcein uptake protocol for assessing staphylococcal channel gating.

Bacterial state	Exposure to	*S. aureus* RN4220 % fluorescent staphylococci	*S. aureus* RN4220 Δ*mscL* % fluorescent staphylococci
Planktonic	Calcein → Formaldehyde	3 ± 4	7 ± 7
Planktonic	Formaldehyde → Calcein	9 ± 8	13 ± 4
Adhering	Calcein → Formaldehyde	45 ± 11	45 ± 11
Adhering	Formaldehyde → Calcein	6 ± 3	9 ± 5
Adhering	Gadolinium → Calcein	9 ± 3	6 ± 2

Fluorescence of planktonic and staphylococci adhering to polystyrene wells due to uptake of calcein prior to and after exposure of bacteria to formaldehyde^33^ or gadolinium to induce channel closure^34,37–39,50^. ± signs represent standard deviations over nine different fluorescence images obtained from three different bacterial cultures.

**Fig. 2:**
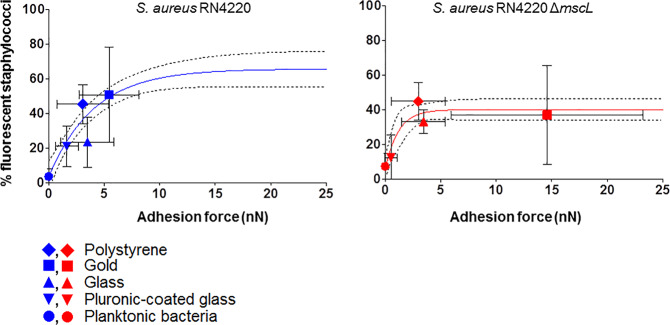
Staphylococcal uptake of calcein. Percentage fluorescent planktonic and adhering staphylococci due to uptake of fluorescent calcein as a function of adhesion force. Lines indicate an exponential fit used to derive plateau levels and critical adhesion forces. Percentage fluorescent staphylococci were calculated with respect to the total number of planktonic bacteria or bacteria adhering to each substratum surface. Dashed lines indicate the 95% confidence band. Horizontal error bars represent standard deviations over at least 45 force–distance curves, comprising for one probe three different spots recording five force–distance curves in each spot. Three probes prepared from three separate bacterial cultures. Vertical error bars represent standard deviations over nine different fluorescence images obtained from three different bacterial cultures.

Considering planktonic staphylococci as bacteria that experience a zero adhesion force, it can be seen in Fig. 2 the percentage bacteria becoming fluorescent due to uptake of calcein in suspension or adhering to the different substratum surfaces. For *S. aureus* RN4220 the percentage fluorescent bacteria increased with the adhesion force exerted by the substratum surface on the adhering bacteria, but for the Δ*mscL* mutant above an adhesion force of 3 nN, no additional uptake of calcein was observed. An exponential fit of the data yielded plateau levels of fluorescent staphylococci that differed for the parent strain and the Δ*mscL* mutant. Plateau levels of calcein fluorescence were achieved at different critical adhesion forces (Table 4). The parent strain achieved a higher plateau level of fluorescent bacteria (66%) at a higher critical adhesion force (4.0 nN) than the Δ*mscL* mutant, reaching a significantly (*p* = 0.0007) lower level of 40% at a significantly (*p* = 0.0149) lower critical adhesion force. This suggests that in absence of large channels in the Δ*mscL* mutant, small channels open at a critical adhesion force of 1.2 nN, while larger channels in the parent strain open and start to contribute to transmembrane transport at a critical adhesion force of 4.0 nN.

**Table 4:** Plateau uptake levels and critical adhesion forces derived from exponentially fitting the relation between bond-matured adhesion forces and uptake of calcein and dihydrostreptomycin.

	*S. aureus* RN4220	*S. aureus* RN4220 Δ*mscL*	*t*, df
Calcein uptake			
Plateau fluorescence (%)	66 ± 16	40 ± 9***	4.166, 16
Critical force *F*_c_ (nN)	4.0 ± 2.5	1.2 ± 1.8*	2.727, 16
Dihydrostreptomycin uptake			
Plateau killing (ΔLog CFU mL^−1^)	−2.3 ± 0.4	−1.0 ± 0.2**	5.035, 4
Critical force (nN)	3.5 ± 5.3	Independent of adhesion force	–

Plateau levels for the percentage of fluorescent staphylococci after calcein uptake and the CFU reduction due to dihydrostreptomycin uptake and critical adhesion forces for each of the two staphylococcal strains used. ± signs represent standard deviations over nine different fluorescence images obtained from three different bacterial cultures for calcein uptake, and three measurements from three different bacterial cultures for dihydrostreptomycin uptake. * indicates significant differences from *S. aureus* RN4220 (two-tailed Student’s *t*-test, *p < 0.05, **p < 0.01, ***p < 0.001). *t*-values and degrees of freedom (df) of the Student’s *t*-test are indicated in the last column.

### Bacterial susceptibility to dihydrostreptomycin and channel gating in adhering staphylococci

**Fig. 3:**
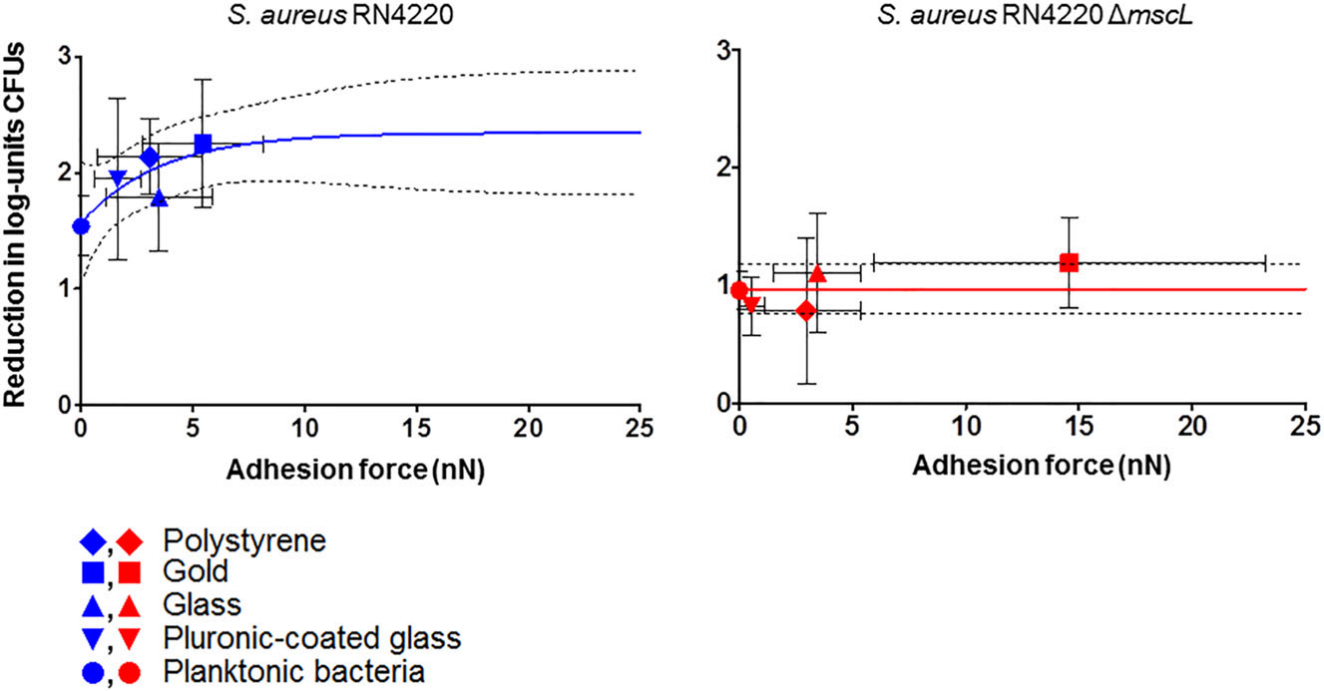
Staphylococcal uptake of dihydrostreptomycin. Reduction in CFUs (log mL^−1^) of planktonic and adhering staphylococci after 2 h exposure to dihydrostreptomycin, expressed relative to exposure to PBS, as a function of adhesion force. Lines indicate an exponential fit used to derive plateau levels and critical adhesion forces. Dashed lines indicate the 95% confidence band. Horizontal error bars represent standard deviations over at least 45 force–distance curves, for one probe comprising three different spots recording five force–distance curves on each spot. Three probes prepared from three separate bacterial cultures. Vertical error bars represent standard deviations over three measurements from three different bacterial cultures.

To evaluate the bacterial uptake of dihydrostreptomycin through mechanosensitive channels, adhering and planktonic staphylococci were exposed to 512 μg mL^−1^ of dihydrostreptomycin for 2 h, well above the minimal inhibitory (MIC) and bactericidal (MBC) concentrations of dihydrostreptomycin determined for *S. aureus* RN4220 (MIC and MBC were both 8 μg mL^−1^) and *S. aureus* RN4220 Δ*mscL* (MIC and MBC were both 16 μg mL^−1^). High concentrations of dihydrostreptomycin were applied to provoke a fast reduction in the number of viable staphylococci. The reduction in the number of CFUs per mL in planktonic staphylococci due to antibiotic exposure was directly compared to a buffer control, while adhering bacteria were dispersed immediately after antibiotic or buffer exposure before CFU determination and calculation of the reduction in the numbers of CFUs achieved. Generally, the parent strain *S. aureus* RN4220 was affected one log-unit more than the Δ*mscL* mutant regardless of the substratum surface (see Supplementary Table 1). Similar to staphylococcal fluorescence due to calcein uptake, staphylococcal killing due to dihydrostreptomycin uptake also increased with adhesion force (Fig. 3). The parent strain exhibited a significantly different behavior than the mutant strain. In the parent strain an exponential increase with adhesion force in staphylococcal killing due to dihydrostreptomycin uptake was observed and after fitting, while a stationary CFU reduction of 2.3 log-units was achieved. This was significantly (*p* = 0.0073) more than the killing observed in the Δ*mscL* mutant (1.0 log-units) that appeared independent of the forces by which they adhered. Interestingly, the critical adhesion force with respect to dihydrostreptomycin killing in the parent strain (3.5 nN) was similar to the critical adhesion force derived from calcein uptake (Fig. 3 and Table 4).

## Discussion

This paper demonstrates that mechanosensitive channels in *S. aureus* are not only opened by changes in membrane tension due to osmotic forces, but also by the forces involved in their adhesion to surfaces. Both types of forces likely work in concert in adhering bacteria. Transmembrane transport of both calcein and dihydrostreptomycin was relatively low in the Δ*mscL* mutant, presumably due to absence of large channels. In the parent strain, transmembrane transport increased when staphylococci adhered with an adhesion force above a critical value of between 3.5 and 4.0 nN, which can be attributed to opening of large channels. Transmembrane transport through physically different small channels levels off toward 1.2 nN. Translation of critical adhesion forces into critical membrane tension changes is impossible, because the necessary rigidity data of the cell wall are not available. However, in planktonic *E. coli* and *S. aureus*, the critical gating membrane tension for MscS was two-fold smaller than for MscL^41^ which is in line with the higher critical adhesion force observed for MscL gating in *S. aureus* as compared with MscS gating^27^. This confirms our hypothesis that adhesion forces play an important mechano-microbiological^42^ role in gating of mechanosensitive channels and suggests that they could play a role in surface sensing.

**Fig. 4:**
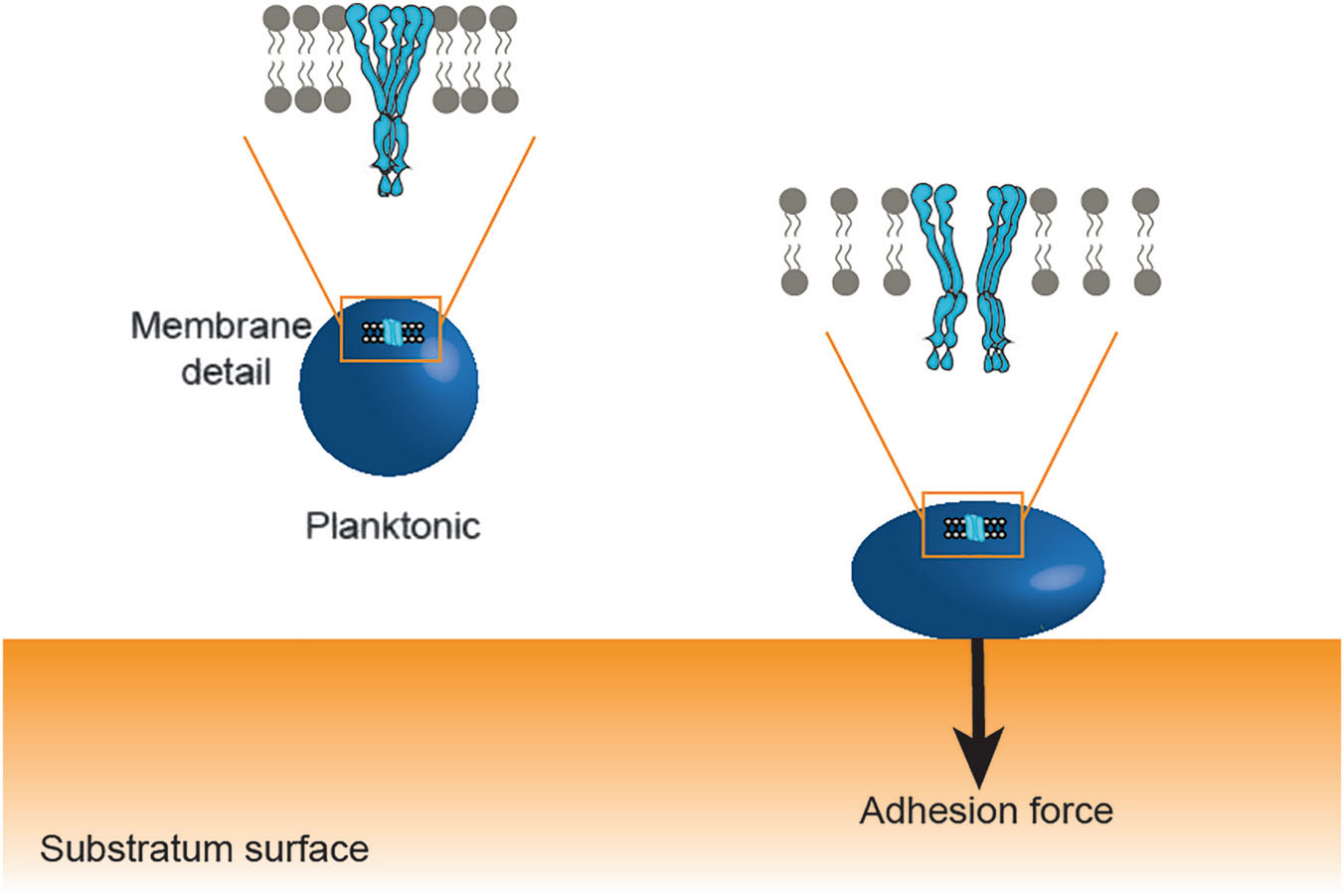
Schematic presentation of the effect of adhesion and adhesion forces on the bacterial cell wall and mechanosensitive channel gating. Planktonic bacteria in the absence of osmotic stress do not experience adhesion forces causing cell wall deformation and changes lipid membrane tension that are accompanied by opening of mechanosensitive channels.

Adhesion forces modulate mechanosensitive channel gating by provoking deformation of staphylococcal cell wall upon bacterial adhesion on a surface. Stronger adhesion forces, such as interactions by force-sensitive adhesins ClfA and ClfB^43^, generate larger cell wall deformation^44^. As a component of the cell wall, the cytoplasmic lipid membrane is also deformed, and accordingly the density of lipids in the membrane decreases^22^, producing a tension change in the membrane that triggers mechanosensitive channel opening^25,45^ (Fig. 4). Gating in reconstituted channels in liposomes^14^ or spheroplasts^27^ depended on membrane properties like membrane thickness^10,25^, stiffness^27^, curvature^23^, and type of lipids^10,24^. Interestingly, the range of force values observed over the different substratum surfaces after bond-maturation was relatively small in *S. aureus* RN4220, while being larger in the Δ*mscL* mutant (see Table 2). Speculatively, this suggests that due to the absence of MscLs in the membrane of the Δ*mscL* mutant, cell wall rigidity and its opposition against deformation and associated membrane tension changes.

Collectively, our results suggest that mechanosensitive channel opening increases with increasing adhesion forces, that were varied in this work by allowing the staphylococci to adhere to different substratum surfaces (Fig. 2). Staphylococcal adhesion forces were stronger on hydrophobic surfaces than on hydrophilic surfaces due to hydrophobic interactions with hydrophobic groups located on the outermost cell surface, such as proteins^46^, fatty acids^47^, or eDNA^48^. In infectious biofilms, bacteria experience adhesion forces from a wide variety of other surfaces, such as of other bacteria, tissue cells or implanted biomaterials. We believe that the role of adhesion forces experienced by the staphylococci in channel gating may be extrapolated to other Gram-positive bacteria. However, due to the role peptidoglycan plays to counter deformation caused by adhesion forces, it is unlikely that these results may be extrapolated to Gram-negative bacteria with a considerably thinner peptidoglycan layer than Gram-positive ones. In conclusion, it is forwarded that channel gating in *S. aureus* is not solely triggered by osmotic forces^5–7^ that are highly different in nature than adhesion forces bacteria experience when adhering to surfaces as in infectious biofilms. As an interesting finding of this work, the adhesion forces experienced by bacteria play a role in mechanosensitive channel gating in the important human pathogen, *S. aureus*, likely in concert with other established driving forces for channel gating.

## Methods

### Bacterial strains and growth conditions

*S. aureus* RN4220 and its isogenic Δ*mscL* mutant (kindly provided by Dr. Jan M. van Dijl, University Medical Center Groningen, The Netherlands) were grown on blood agar plates at 37 °C for 24 h. A single colony was used to inoculate 10 mL of Tryptone Soya Broth (TSB; OXOID, Basingstoke, UK), incubated at 37 °C for 24 h. This preculture was then diluted 1:20 in 100 mL of fresh TSB and incubated at 37 °C for 16 h.

Cultures were harvested by centrifugation (5000 × *g*) and washed twice in phosphate buffered saline (PBS: 5 mM K_2_HPO_4_, 5 mM KH_2_PO_4_, 0.15 M NaCl, pH 7.0). Staphylococci were subsequently resuspended in PBS and sonicated (3 × 10 s, 30 W) in an ice-water bath (Vibra Cell Model 375, Sonics and Materials Inc., Danbury, CT, USA). The bacterial suspension was diluted in PBS to a concentration of 10^8^ mL^−1^ as determined in a Bürker-Türk counting chamber. Absence of *mscL* genes in the mutant strain was verified by DNA sequencing using Illumina MiSeq as previously described^49^ (Supplementary Fig. 1).

### Materials preparation and characterization

Polystyrene from 12-well-culture-plates (Greiner Bio-One, Frickenhausen, Germany) and gold-coated (10 nm thickness) glass slides (DLRI, St. Charles, MO, USA) were used as received after extensive rinsing with demineralized water. Borosilicate glass (Menzel-Gläser, Menzel GmbH&Co KG, Braunschweig, Germany) was cleaned with 2% Extran, 5 min sonication with 2% RBS35, methanol, and demineralized water. For creating polymer-brush-like surfaces, clean glass slides were exposed to a solution of 0.5 g L^−1^ Pluronic F-127 (PEO_99_PPO_65_PEO_99_, molecular weight 12600; Sigma-Aldrich, St. Louis, MO, USA) in demineralized water for 20 min. Gentle rinsing with demineralized water removed non-attached Pluronic F-127. Coupons of 1 cm^2^ were prepared to fit into 12-well plates.

Surface roughnesses were measured with an atomic force microscope (AFM; BioScope Catalyst, Bruker, Camarillo, CA, USA), using ScanAsyst-air tips (tip curvature radius 2 nm; Bruker) to scan areas of 50 × 50 μm at a rate of 1 Hz. Contact angles were measured with water, formamide and methylene iodide, using the sessile drop technique and a home-made contour monitor. Contact angles with these liquids, having different surface tensions and polarities, allowed calculation of the total surface free energy (γ^tot^), together with its acid-base (γ^AB^) component, which in turn can be divided into electron-donating (γ^−^) and -accepting (γ^+^) parameters, and the Lifshitz–Van der Waals (γ^LW^) component^50^. Surface roughness and contact angles were measured in triplicate on three different substratum surfaces.

### Contact angles on bacterial lawns and surface free energies

Hydrophobicity of bacterial cell surfaces was determined through contact angle measurements with different liquids on staphylococcal lawns using the sessile drop technique and a home-made contour monitor. Staphylococci were deposited on 0.45-μm pore-size HA membrane filters (Millipore Corporation, Bedford, MA, USA) using negative pressure, and filters were subsequently dried until reaching constant, so-called “plateau” water contact angles, representing bacterial cell surfaces without “free”, but with “bound” water. Contact angles were measured with water, formamide and methylene iodide on six bacterial lawns from three different bacterial cultures, from which surface free energy components and parameters were then calculated as described in the above section.

### Zeta potentials

To measure bacterial zeta potentials, bacteria were resuspended in 10 mM potassium phosphate buffer at different pH values (pH 2, 3, 4, 5, 7). Using the Helmholtz–Smoluchowski equation^51^, zeta potentials were derived from electrophoretic mobilities obtained with particulate microelectrophoresis (Zetasizer Nano ZS, Malvern Instruments, Worcestershire, UK). Experiments were performed in triplicate with different bacterial cultures.

### Microbial adhesion to hydrocarbons (kinetic MATH assay)

The combined effects of surface hydrophobicity and charge on staphylococcal adhesion to a hydrophobic ligand were determined, as previously described^52^. Briefly, staphylococci were resuspended in 3 mL of 10 mM potassium phosphate buffer containing 1:20 (v/v) hexadecane at different pH values (pH 2, 3, 4, 5, 7) to an optical density at 600 nm between 0.4 and 0.6 (initial absorbance at time zero [*A*_0_]), as spectrophotometrically measured (Spectronic 20 Genesys, Spectronic Instruments, Rochester, NY, USA). The suspension was vortexed for 10 s and allowed to settle for 10 min, and the optical density was measured again (absorbance at time *t* [*A*_*t*_]). This procedure was repeated for five more times to enable calculation of an initial rate of bacterial removal from the aqueous phase defined as

1 $${\mathrm{Rate}}\,{\mathrm{of}}\,{\mathrm{initial}}\,{\mathrm{removal}} = \mathop {{\lim }}\limits_{t \to 0} \frac{{\mathrm{d}}}{{{\mathrm{d}}t}}\log \left( {\frac{{A_t}}{{A_0}} \times 100} \right)$$

where *t* is the vortexing time. The experiment was performed in triplicate with different bacterial cultures.

### Atomic force microscopy

Single-bacterial contact AFM probes were prepared by immobilizing bacteria on NP-O10 tip-less cantilevers (Bruker), as described previously^53^. Briefly, cantilevers were calibrated by the thermal tuning method displaying spring constants in range 0.03–0.12 N m^−1^ and mounted on a micromanipulator (Narishige International, Tokyo, Japan) under microscopic observation (Leica DMIL, Wetzlar, Germany). The cantilever apex was then dipped into a droplet of 0.01% poly-l-lysine (molecular weight 70,000–150,000, Sigma-Aldrich) for 1 min, dried in air for 2 min and dipped into a bacterial suspension droplet (3 × 10^6^ mL^−1^ in 10 mM potassium phosphate buffer, pH 7.0) for 2 min. Imaging a calibration grid (HS-20MG BudgetSensors, Innovative Solutions Bulgaria Ltd., Sofia, Bulgaria) with the bacterial probe confirmed single-bacterial contact with the substratum surface^54^, and probes yielding double contour lines were discarded (which seldom or never happened).

AFM force measurements were done on a BioScope Catalyst AFM (Bruker), at room temperature in 10 mM potassium phosphate buffer. Force–distance curves were obtained under a loading force 3 nN at approach and retraction velocity 2 μm s^−1^, and taken prior to and after 10 s bond-maturation. To verify that measurements did not disrupt bacterial integrity, five force–distance curves at a loading force of 3 nN and 0 s contact time were acquired on clean glass at the onset and end of each experiment. When adhesion forces differed more than 1 nN from the onset to the end of an experiment, the probe and its last data set obtained were discarded and the probe was replaced by a new one. For each strain, AFM measurements were performed with at least three probes prepared from three separate bacterial cultures. With each probe, at least three different spots on each substratum surface were measured, recording five force–distance curves on each spot for each contact time.

### Calcein uptake

For measuring the gating of mechanosensitive channels, bacterial uptake of the fluorescent dye calcein (Sigma-Aldrich) was monitored. First, bacteria were allowed to sediment from a suspension in PBS (10^8^ mL^−1^) onto the different substratum surfaces for 90 min, after which PBS was removed and gentle rinsing with PBS was applied to remove non-adhering bacteria. Subsequently, calcein was added in a concentration of 4 mM for 15 min, followed by fixation with 4% formaldehyde (VWR International, Breda, Netherlands) for 15 min to prevent removal of intracellular calcein. Extensive rinsing in ultrapure water removed the excess extracellular calcein.

As a control, planktonic bacteria were used. Calcein (4 mM) was added to a staphylococcal suspension for 15 min, followed by fixation with 4% formaldehyde for 15 min. Excess extracellular calcein was removed by filtration with 0.45-μm pore-size HA membrane filters (Millipore Corporation). Bacteria were then collected from the filters by vortexing and resuspended in PBS.

Phase-contrast and fluorescent images were acquired with a fluorescence microscope (Leica, Wetzlar, Germany) on both adhering and planktonic bacteria, to evaluate total number of adhering bacteria or in suspension and the percentage of bacteria that displayed calcein uptake. The percentage of bacteria that displayed fluorescence in suspension or adhering to a surface was taken as a measure for calcein uptake, according to a protocol previously described for gating in mechanosensitive channels reconstituted in liposomes^33^ and validated here for use in bacteria (Table 3). For validation, channels were blocked by 15-min exposure to 4% formaldehyde^34^ or blocking was induced by 15-min exposure to 100 μM gadolinium^55^ before calcein exposure.

The increase in the percentage fluorescent staphylococci (*Y*) as a function of their adhesion forces (*F*) on the different substratum surfaces was fitted to an exponential function to yield a plateau level of the percentage of fluorescent staphylococci and a critical adhesion force according to

2 $$Y = Y_0 + \left( {Y_{{\mathrm{plateau}}} - Y_0} \right)\left( {1 - e^{ - \frac{F}{{F_{\mathrm{c}}}}}} \right)$$

in which *Y*_0_ is the percentage fluorescent staphylococci in suspension (zero adhesion force) and *F*_c_ is the critical adhesion force. *Y*_plateau_ is the plateau level of the percentage fluorescent staphylococci reached.

To allow more accurate fitting, the plateau level was mathematically confined to the maximum percentage of adhering, fluorescent staphylococci observed for each strain. Experiments were performed in triplicate with different bacterial cultures.

### Dihydrostreptomycin susceptibility and uptake

First, MIC and MBC of the staphylococci for dihydrostreptomycin were determined. To this end, bacterial cultures (10^5^ mL^−1^ in TSB) were dispensed in a 96-well microtiter plate with dihydrostreptomycin sesquisulfate (Sigma-Aldrich) in TSB with known concentrations and applying a step factor dilution of 2 starting from 512 μg mL^−1^. Incubation was done at 37 °C for 24 h. After incubation, MIC was taken as the lowest antibiotic concentration not generating visible turbidity. Then, 10 μL of bacterial suspensions of each well showing no turbidity, were plated on TSB agar plates and incubated at 37 °C for 24 h. The MBC was taken as the lowest concentration at which no colonies were visible on the plate. Experiments were performed in triplicate with different bacterial cultures.

To evaluate the differential uptake of dihydrostreptomycin through mechanosensitive channels^15^ in adhering and planktonic staphylococci, bacterial suspensions (10^8^ mL^−1^ in PBS) were allowed to sediment on different surfaces for 90 min or maintained planktonically in suspension. After subsequent exposure to dihydrostreptomycin (512 μg mL^−1^) in PBS for 120 min, bacteria were collected by 1 min sonication in a water bath and serially diluted in PBS. Exposure to PBS was applied as a control. Next, 10-μL aliquots were plated on TSB agar plates and incubated for 16 h at 37 °C. The number of colonies formed on the plates was manually counted. Experiments were performed in triplicate with different bacterial cultures.

The reduction in the number of CFUs per mL as a function of their adhesion forces, *F*, on the different substratum surfaces was fitted to an exponential function to yield a plateau level of the reduction in the number of CFUs per mL and a critical adhesion force, similar as done for the percentage fluorescent staphylococci (Eq. (2)). To allow more accurate fitting, the plateau level was mathematically confined to the maximum percentage of reduction in the number of CFUs per mL observed for each strain. Experiments were performed in triplicate with different bacterial cultures.

### Statistical analysis

GraphPad Prism, version 8.4.3 (San Diego, CA, USA) was employed for statistical analysis. Data were tested for normal distribution with Shapiro–Wilk normality test. If data were normally distributed, one-way analyses of variance (ANOVA) with Tukey’s HSD post hoc test or a two-tailed Student’s *t*-test were employed. When data were not normally distributed, Kruskal–Wallis test with Dunns’ approximation replaced ANOVA. Comparisons of dihydrostreptomycin killing across substratum surfaces between the parent strain and its mutant were made using one-way ANOVA test with Sidak’s multiple comparison adjustment (four comparisons). *p* < 0.05 was used as significance for all tests.

## Supplementary information

Supplementary Information

## Data Availability

The authors declare that the data supporting the findings of this study are available within the paper and its Supplementary information files.
