# Supplementary material for: Role of adhesion forces in mechanosensitive channel gating in Staphylococcus aureus adhering to surfaces
Source: NPJ Biofilms Microbiomes. 2020 Aug 21;6:31. doi: 10.1038/s41522-020-00141-z (PMC7442641; doi:10.1038/s41522-020-00141-z)
Supplement: Supplementary file 1 — Supplementary Information [file 41522_2020_141_MOESM1_ESM.pdf]

Supplementary Information

Role of adhesion forces in mechanosensitive channel gating in *Staphylococcus aureus* adhering to surfaces

Vera Carniello, Brandon W. Peterson*, Henny C. van der Mei, Henk J. Busscher

University of Groningen and University Medical Center Groningen, Department of BioMedical Engineering, Groningen, Netherlands

*Corresponding author:

Brandon W. Peterson

Department of Biomedical Engineering-FB40

University Medical Center Groningen

Antonius Deusinglaan 1

9713 AV Groningen

The Netherlands

Tel +31 50 3616110

b.w.peterson@umcg.nl

Supplementary Table 1

Staphylococcal uptake of dihydrostreptomycin on different substratum surfaces

CFU reduction (ΔLog CFU mL^{-1}) as due to dihydrostreptomycin uptake on different substratum surfaces compared with PBS exposure. ± signs represent standard deviations over three measurements from three different bacterial cultures for dihydrostreptomycin uptake. * indicates significant differences from *S. aureus* RN4220 (One-way ANOVA, Sidak, ** $p < 0.01$). t-values and degrees of freedom (df) of the One-way ANOVA with Sidak's multiple comparison correction (4 comparisons) are indicated in the last column.

	***S. aureus* RN4220**	***S. aureus* RN4220 Δ*mscL***	**t, df**
**Polystyrene**	-2.1 ± 0.3	-0.8 ± 0.6**	3.525, 18
**Gold**	-2.3 ± 0.6	-1.2 ± 0.4	2.589, 18
**Glass**	-1.8 ± 0.5	-1.1 ± 0.5	1.792, 18
**Pluronic-coated glass**	-1.9 ± 0.7	-0.8 ± -0.3	2.735, 18

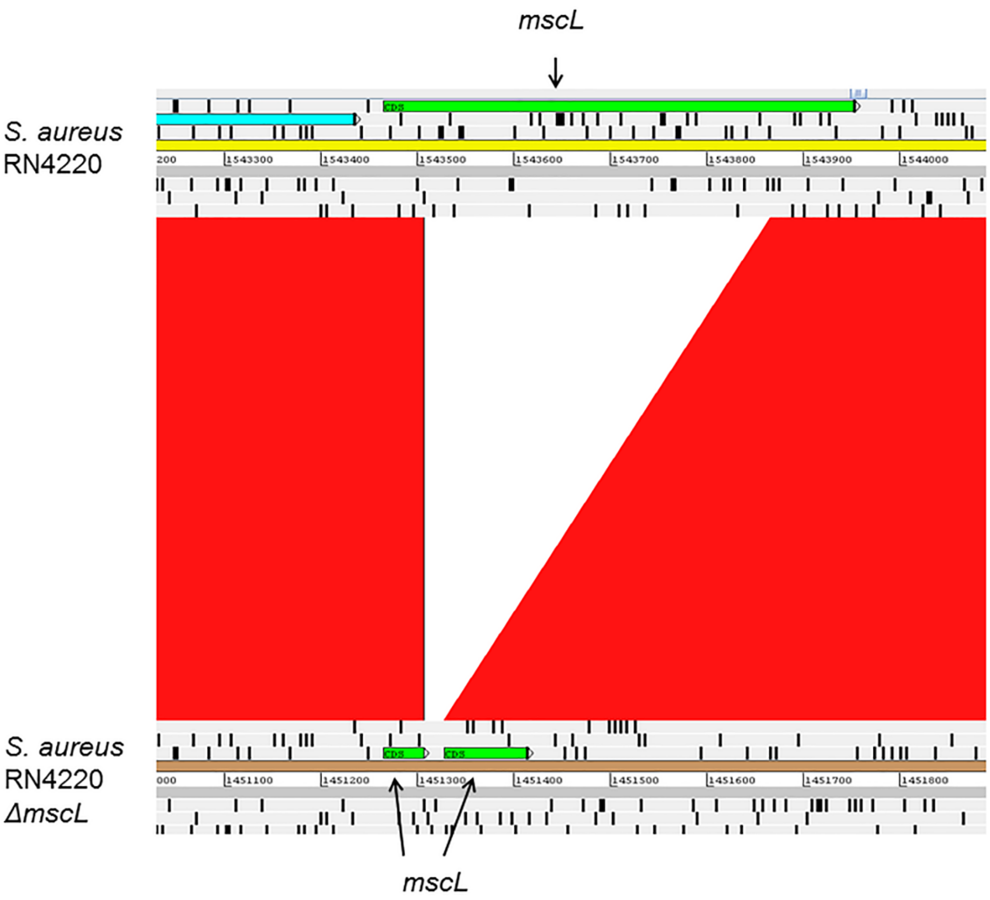

Supplementary Figure 1. DNA sequencing of mscL genes in the parent *S. aureus* RN4220 strain and its isogenic *ΔmscL* mutant. Absence of mscL genes in the *ΔmscL* mutant strain verified by DNA sequencing. Pair-wise comparison of *S. aureus* RN4220 (upper sequence) and *S. aureus* RN4220 *ΔmscL* (lower sequence). Red areas represent regions with BLASTN matches of 99.9% in the same orientations, visualized with Artemis Comparison Tool.[1,2]

REFERENCES

1. Carver, T. J. *et al*. ACT: the Artemis comparison tool. *Bioinformatics* **21**, 3422-3423 (2005).

2. Desmet, S. *et al*. Antibiotic resistance plasmids cointegrated into a megaplasmid harboring the bla OXA-427 carbapenemase gene. *Antimicro. Agents Chemother*. **62**, e01448-17 (2018).
